# Capturing the patient experience in systemic lupus erythematosus: Are widely used measures fit-for-purpose and adherent to FDA PRO guidance recommendations?

**DOI:** 10.1186/s41687-022-00411-8

**Published:** 2022-01-21

**Authors:** Kayleigh R. Majercak, Eleanor M. Perfetto, Ester Villalonga-Olives

**Affiliations:** 1 Department of Pharmaceutical Health Services Research, School of Pharmacy, University of Maryland Baltimore, 220 Arch Street, 12th Floor, Baltimore, MD 21201 USA; 2 National Health Council, Washington, DC USA

**Keywords:** Patient-reported outcome, Systemic lupus erythematosus, Clinical outcome assessments, FDA PRO guidance, Fit-for-purpose, Patient experience

## Abstract

**Background:**

The 2009 Food and Drug Administration (FDA) patient-reported outcome (PRO) guidance outlines characteristics of rigorous PRO-measure development. There are a number of widely used PRO measures for Systemic Lupus Erythematosus (SLE), but it is unknown how well the development processes of SLE PRO measures align with FDA guidance; including updated versions. The objective of this study was to assess how well the LupusQoL and LupusPRO, and corresponding updated versions, LupusQoL-US and LupusPROv1.8, align with Food and Drug Administration (FDA) 2009 patient-reported outcome (PRO) guidance.

**Methods:**

LupusQoL and LupusPRO were selected as the most widely studied and used Lupus PROs in the UK and US. Original (LupusQoL (2007) and LupusQoL-US (2010)) and revised (LupusPROVv1.7 (2012) and LupusPROv1.8 (2018)) versions were reviewed. We used FDA PRO guidance to create evaluation criteria for key components: target population, concepts measured, measurement properties, documentation across the phases of content validity (item-generation and cognitive interviewing, separately) and other psychometric-property testing. Two reviewers abstracted data independently, compared results, and resolved discrepancies.

**Results:**

For all measures, the target population was unclear as population characteristics (e.g., ethnicity, education, disease severity) varied, and/or were not consistently reported or not considered across the three phases (e.g., LupusQoL item-generation lacked male involvement, LupusPRO cognitive-interviewing population characteristics were not reported). The item-generation phase for both original measures was conducted with concepts elicited via patient-engagement interviews and item derivation from experts. Cognitive interviewing was conducted via patient feedback with limited item-tracking for original measures. In contrast, the revised measures assumed content validity. Other psychometric testing recommendations (reliability, construct validity, ability to detect change) were reported for both original and revised measures, except for ability to detect change for revised measures.

**Conclusions:**

The SLE PRO measures adhere to some but not all FDA PRO guidance recommendations. Limitations in processes and documentation of the study population, make it unclear for which target population(s) the current Lupus measures are fit-for-purpose.

**Supplementary Information:**

The online version contains supplementary material available at 10.1186/s41687-022-00411-8.

## Introduction

During phase III clinical trials, the effect of an intervention is assessed by comparing differences in endpoints between the intervention and control groups to determine if the intervention provides treatment benefit. A “treatment benefit” is defined as “a favorable effect on a meaningful aspect of how a patient feels or functions in his or her life, or on his or her survival” [1]. It may be evaluated using tools that directly or indirectly measure how patients feel, function, or survive [1, 2]. Direct evidence of treatment benefit is a measure of a meaningful health aspect, such as survival or a direct report from patients regarding feelings and functions in their daily activities living with their condition.^1^ Conventionally, the incorporation of patient-reported outcome (PRO) measures in clinical trials has been relatively low due to lack of measure standardization and use [3]. PRO measures, however, are vital in the evaluation of treatment benefit, especially if survival is not a consideration. Due to increased efforts in understanding patient perspectives and experiences with their respective condition(s) and treatment(s), the application of PROs within drug development has gained substantial momentum [4, 5].

In 2009, the FDA published guidance on use of PRO measures in medical-product development to support labeling claims [6]. This guidance outlines characteristics of rigorous PRO-measure development and provides insight into “FDA’s current thinking” in the evaluation and determination for whether measures are fit-for-purpose [6]. As a result, using the guidance as a reference positions sponsors to maximize the likelihood of success in demonstrating treatment benefit when incorporating PROs in clinical trials. Likewise, the guidance may also be applied for PROs used outside of clinical trials. Following the guidance ensures a higher likelihood PROs are a reflection of the patient voice and outcomes reported are robust and meaningful by using state-of-the-art methods in both the qualitative and quantitative parts of development processes. Furthermore, an emphasis is placed on the concepts measured being comprehensive, relevant, and meaningful to patients (i.e., evidence of content validity). The instrument’s authenticity is a product of direct engagement with patients in the qualitative process; however, transparency and documentation are often lacking [7, 8].

Despite the development of the FDA guidance, most PRO measures are not qualified to be used as supporting evidence for approval in the drug-development process. The collected data often fail as endpoints in phase III clinical trials [9]. A study by DeMuro et al. [10], reviewed the rationale behind decisions to reject PRO claims and found that a main concern was the evidence of content validity was lacking (e.g., insufficient documentation of validation in the target population). Similarly, in a recent publication by Hong et al., the authors found that none of the PRO data collected in clinical trials for approved breast cancer treatments from 2000 to 2019 were included in the drug product labeling. The PRO data was deemed unacceptable due to “lack of meaningfulness and clinical significance, lack of content validity, and inadequate analytical methods” [11]. It is important to note, a new FDA patient-focused drug development (PFDD) guidance series is underway to provide more detail and clarity on the use of COAs for regulatory approval of medical products. With draft and final releases of the FDA PFDD guidance series still pending, the 2009 guidance remains in effect, though it will be replaced when the new guidances are released.

Systemic lupus erythematosus (SLE) is a chronic, inflammatory, autoimmune condition that can affect multiple organ systems [12, 13]. Survival is no longer the primary concern of individuals diagnosed with SLE as 10-year survival has significantly improved up to 91.4% [14]. As a result, assessments of patients with SLE has shifted focus from survival to health-related quality of life (HRQoL), “a multi-domain concept that represents the patients’ general perception of the effect of illness and treatment on physical, psychological, and social aspects of life” [6].

The two most-widely used lupus PRO tools are the LupusQoL and the LupusPRO. The LupusQoL, a HRQoL condition-specific PRO for lupus, was developed in the UK and published in 2007 [15]. Due to linguistic and cultural differences, Jolly et al. culturally adapted and psychometrically assessed the instrument in 2010 to be used in the ethnically heterogeneous SLE population in the United States (US) [16]. As a consequence of the limited generalizability of the instrument to patients with SLE in the United States, the LupusPRO v1.7 became available in 2012 and was revised in 2018 (v1.8) [17, 18]. Two systematic reviews [19, 20] and one review [21] published between 2018 and 2021 highlight the use of LupusQoL and LupusPRO instruments. LupusQoL has been used in three drug-related RCTs, however the collected data were used in PRO exploratory analyses [22, 23]. LupusPRO has not been used in RCTs, only pilot studies of health interventions [24, 25].

As these two most widely used PRO measures for SLE were developed prior to and just after the release of the FDA PRO guidance, it is unknown how well the development process of earlier instruments and respective updated versions align with FDA PRO guidance recommendations. It would be expected that those released after 2009 would be more likely to adhere to guidance recommendations. Importantly, the effective use of the guidance may enhance standardization of the process and documentation, thereby raising uptake in the use of newer PRO measures. The objective of this study was to assess how well the two widely used SLE PRO measures, the LupusQoL and LupusPRO, and corresponding updated versions, LupusQoL-US and LupusPROv1.8, align with FDA guidance.

## Methods

### SLE-measure selection

Using the literature [19, 26, 27] to guide SLE-measure selection, it was determined that LupusQoL and LupusPRO are the most widely used measures for lupus. Both measures are consistently cited in reviews [19–21, 26, 27], are among the few available SLE-measures developed using patient input [26] and were extensively studied with more than three validation publications in English-speaking SLE populations [19]. Thus, they were well suited for this exercise.

We conducted a methodological review of the SLE PRO measures in the UK and US. Four versions were reviewed: The two original (LupusQoL (2007) and LupusPROVv1.7 (2012)) and the two revised (LupusQoL-US (2010) and LupusPROv1.8 (2018)). Throughout the paper, we will refer to LupusQoL and LupusPROv1.7 as original measures and LupusQoL-US and LupusPROv1.8 as revised measures. Brief summaries of the instruments are provided in the Additional file 1: Appendix 2. Publications for original and revised measures, describing development and psychometric assessment were identified using PubMed. Publications addressing additional testing of the measures were included as well, e.g., additional measurement properties not previously tested. As PubMed provided original, revised, and secondary testing publications, supplementary databases were not﻿ deemed necessary. PRO review articles also were scanned to ensure all relevant publications were assessed [19–21, 27–30]. The methodological analysis sought information describing the methods and processes employed for instrument development and testing of the original and revised lupus instruments.

### Evaluation criteria for PRO measures

The 2009 FDA PRO guidance describes the PRO instrument-development framework. To develop evaluation criteria, we focused on this framework’s sequential process of content validity (item generation, cognitive interviewing), testing of other psychometric properties, and measure modifications [6].

The developed evaluation criteria examined key components (target population, concepts measured, measurement properties, and documentation) across all phases (item generation, cognitive interviewing, and testing of other psychometric properties). The operationalization of the evaluation criteria is presented in Table 1 with corresponding instructions for use. Briefly, a user does not need to answer all questions if a previous response is indicated as “No”, especially for questions with an asterisk. Further, if the response to a question with an asterisk * is “No” for target population, concepts measured, or measurement properties, content validity is deemed questionable. As a result, additional testing (e.g., other psychometric property testing) is irrelevant and evaluation of other key concepts, i.e., measurement properties and documentation is not appropriate. For the purposes of the study, the evaluation was carried out for all key components. To support understanding of the criteria, Additional file 2: Table S1 provides FDA PRO Guidance terms and definitions used. Additional file 3: Table S2 showcases an overview of the assessment content based upon the evaluation criteria table (Table 1).

**Table 1:** Evaluation criteria for key components of patient-reported outcome measure (PRO) measure development and testing

Target population		Yes/no
1.*	*Is the target population transparent?* Clear reporting of the decisions used in the study regarding selection of the target population; defines the intended population the researchers targeted to generalize results of PRO development and testing	
2.*	*Is the target population clearly specified?* Sufficient details are provided about the intended population to understand the generalizability of the study as well as the study population that should be targeted for PRO development and testing	
3.*	*Are the study-population characteristics reported for all phases of development?* Study characteristics are reported in item generation, cognitive interviewing, and other psychometric property testing	
4.*	*Are relevant study-population characteristics (age, sex, condition severity, race, *etc*.) explicitly identified for the phases of development conducted?* All the important demographic/clinical characteristics are reported for each phase. This may be based upon objectives, inclusion/exclusion criteria, etc. that indicate the target population	
5.*	*Are the study-population characteristics reported consistent across all phases of development?* The percentage of participants for each characteristic are similar across phases	
6	*Are minimum recruitment targets for the study population stated to ensure the study population is representative of the target population of interest for each phase?* Sample size targets are provided for demographic/clinical characteristics	
Concepts measured		Yes/no
7.*	*Is the approach for derivation of items appropriate for item generation?* Using patient interviews/focus groups, open-ended questions during interviews	
8.*	*Is the approach for derivation of items comprehensive for item generation?* Identifying concepts/items that matter most to patients including symptoms and/or activities experienced by the majority of the patients representing the target population There is evidence that interviews/focus groups encompassed a wide range of patients representing the target population	
9.*	Was cognitive interviewing conducted via patient input?	
10.*	Was the feedback collected during cognitive interviewing considered and, if not, was an explanation provided for why feedback was not incorporated?	
11	*Was quantitative testing of items/concepts conducted?* Testing of other psychometric properties (e.g., reliability, construct validity, etc.)	
Measurement properties		Yes/no
12.*	*Is there evidence that saturation was achieved during item generation?* No new relevant information emerges from interviews/focus group discussions	
13.*	*Is there evidence that saturation was achieved during cognitive interviewing?* No new relevant information emerges from interviews/focus group discussions	
14.*	*For each of the relevant study population characteristics identified for the target population, were minimum targets met?* Minimum targets are met, evidence of representativeness, etc	
15	Are reliability results reported?	
16	Are construct-validity results reported?	
17	Are ability-to-detect-change results reported?	
Documentation		Yes/no
18	*Is the study population clearly specified?* Sufficient details are provided about the study population to generate a reproducible study	
19	Is there documentation of elicited concepts (e.g., transcripts, quotes, saturation grid, etc.)?	
20	Is there documentation of cognitive interviewing feedback being incorporated (e.g., stated as revisions made, item-tracking matrix, etc.)?	
21	Are the number of participants for each phase clearly reported, including characteristics of the study population?	
22	*Is there documentation of appropriate content of the measure?* Exact words used by patients to represent concepts, response options, recall period, etc	
23	*Is the item generation-process transparent?* Clear reporting of the decisions used in the study, what the researchers actually did Item generation techniques, theoretical approaches, source of items	
24	*Is the cognitive-interviewing process transparent?* Clear reporting of the decisions used in the study, what the researchers actually did	
25	*Are the quantitative techniques transparent?* Clear reporting of the decisions used in the study, what the researchers actually did. The methods for domain generation, testing of reliability, construct validity, etc	
26	*Are the other psychometric testing results clear?* The number of domains generated, reliability estimates, construct validity results, etc	

*Please see instructions in the Additional file 1: Appendix 1.

### Data abstraction

The criteria guided reviewers on what data to look for to abstract. Identified data were abstracted for each criterion when found. If no data were identified for the criterion, “Not Available” was inserted; thus, the response to the criterion was “No.” The criteria also guided assessment if data identified adhered to the FDA guidance as demonstrated with a response to the criterion as “Yes”. Two reviewers, KM and CS, abstracted and scored data independently, compared results, and resolved discrepancies. A third reviewer (EV or EP) served as a tie breaker.

#### Target population

Detailed information abstracted was deemed sufficient if characteristics of the target population could be identified and documentation of the study population was considered representative of the intended population of interest based upon the response “Yes” to questions 1–6 in the evaluation criteria table (Table 1). Questions with an asterisk were essential for sufficient evidence.

#### Concepts measured

The concepts measured should reflect what is most important to the target population of patients with the condition [6]. Information regarding patient interviews, focus groups, and qualitative cognitive interviewing was abstracted pertaining to items included in the instrument and was evaluated to confirm understanding as well as completeness of the concepts measured. The response “Yes” to questions 7–11 corresponded to sufficient evidence for concepts measured. Questions with an asterisk were essential for sufficient evidence for content validity and continuing the evaluation. Question 11 is required for the other psychometric property testing phase to be sufficient but was not deemed essential to continue the evaluation.

#### Measurement properties

The description of methods and results for measurement properties were assessed to determine if measurement properties included all expected attributes (content validity, construct validity, reliability, and ability to detect change) [6]. This step entailed making sure content validity was deemed adequate and statistical analyses were conducted with the results reported for the testing of other psychometric properties. The measurement properties component was rated using questions 12–17 by a Yes/No format. If any of the questions with an asterisk * are indicated as “No”, the evidence is insufficient for item generation and cognitive interviewing. Questions 15–17 for other psychometric property testing phase are deemed as “Available” or “Not Available” based upon “Yes” or “No” responses, respectively.

#### Documentation

Documentation of the development process is critical. We scored whether or not there was good documentation by looking at questions 18–26 by a Yes/No format. If any of the questions are indicated as “No”, the evidence is insufficient for the corresponding phase of development.

The determination of sufficient/insufficient evidence for key components was synthesized based on “No” responses. Five of the six questions for the target population component were deemed as required as denoted with an asterisk for continuing the evaluation. Likewise, the concepts measured component of the assessment comprised content validity and other psychometric testing phases with questions 7 through 11. Four of the five questions were required to continue with the evaluation as these were related to content validity. The last question in the section pertained to other psychometric property testing and was required for sufficient evidence for that phase of development. Three of the questions contained in measurement properties were denoted with an asterisk *. Lastly, all nine questions were necessary to indicate sufficient evidence for documentation.

## Results

Table 2 provides the study population characteristics of SLE population used in the development and validation of SLE-PRO measures. Data abstraction notes for concepts measured is provided in Table 3. Similarly, data abstraction notes for measurement properties and documentation are summarized in Table 4. An in-depth summary of the evaluation results for target population, concepts measured, measurement properties, and documentation are presented for each SLE-measure separately in the Additional file 1: Appendix 3.

**Table 2:** Study population characteristics of SLE population used in the development and validation of SLE-PRO measures

	LupusQoL (n = 30)	LupusQoL-US (n = N/A)	LupusPRO v1.7 (n = 18)	LupusPRO v1.8 (n = N/A)
*Item generation*^b^				
Female, n (%)	30 (100%)	N/A	16 (88.9%)	N/A
Age in years, mean (SD)	48.1 (13.1)	N/A	45.1 (12.3)	N/A
Race, n (%)				
White	22 (73.3%)	N/A	39%	N/A
Black/African American	0	N/A	39%	N/A
Hispanic	0	N/A	17%	N/A
Asian	8 (26.7%)	N/A	5%	N/A
Other	0	N/A	0	N/A
Education years (SD)	12.7 (4.5)	N/A		N/A
SLE duration in years, mean (SD)	9.2 (8.4)	N/A	9.1 (6.1)	N/A
Disease activity, mean (SD)	N/A	N/A	SLEDAI 6.6 (6.5)	N/A
Disease damage, mean (SD)	N/A	N/A	SDI 2.0 (2.0)	N/A

	(n = 20)	(n = 15)	(n = 70)	(n = N/A)
*Cognitive interviewing*^b^				
Female, n (%)	20 (100%)	N/A	N/A	N/A
Age in years, mean (SD)	52 (15.2)	N/A	N/A	N/A
Race, n (%)				
White	18 (90%)	N/A	N/A	N/A
Black/African American	1 (5%)	N/A	N/A	N/A
Hispanic	0	N/A	N/A	N/A
Asian	1 (5%)	N/A	N/A	N/A
Other	0	N/A	N/A	N/A
Education years (SD)	12.9 (3.3)	N/A	N/A	N/A
SLE duration in years, mean (SD)	11.2 (6.1)	N/A	N/A	N/A
Disease activity, mean (SD)	N/A	N/A	N/A	N/A
Disease damage, mean (SD)	N/A	N/A	N/A	N/A

	(n = 160)	(n = 186)	(n = 323)	(n = 131)^a^
*Other psychometric testing*				
Female, n (%)	152 (95%)	175 (94%)^a^	301 (93%)	120 (91%)
Age in years, mean (SD)	45.3 (10.8)	42.5 (12.9)	43.3 (13.3)	40.4 (14.1)
Race, n (%)				
White	N/A	43 (23%)^a^	75 (23%)^a^	31 (23%)^a^
Black/African American	N/A	112 (60%)^a^	104 (32%)^a^	68 (52%)^a^
Hispanic	N/A	23 (12%)^a^	133 (41%)^a^	17 (13%)^a^
Asian	N/A	11 (6%)^a^	10 (3%)^a^	7 (5%)^a^
Other	N/A	N/A	7 (2%)^a^	11 (8%)^a^
Education < high school, n (%)	N/A	N/A	94 (29%)^a^	6 (4.4%)
SLE duration in years, mean (SD)	N/A	N/A	9.3 (7.6)	8.13 (6.84)
Disease activity, mean (SD)	N/A	SLEDAI 6.2 (5.8)	SLEDAI 3.9 (3.8)	SLEDAI 4.89 (4.43)
Disease damage, mean (SD)	N/A	SLICC 2.0 (2.1)	SDI 0.7 (1.1)	SDI 0.61 (1.05)

N/A, not available; SLEDAI, Systemic Lupus Erythematosus Disease Activity Index; BILAG, British Isles Lupus Assessment Group; SLICC, Systemic Lupus International Collaborating Clinics; SDI, The Systemic Lupus International Collaborating Clinics/American College of Rheumatology (ACR) Damage Index

^a^The counts were derived based on percentages provided in publication, and therefore are approximate.

^b^Content validity encompasses both item generation and cognitive interviewing

**Table 3:** Key points of data abstraction and assessment for concepts measured using evaluation criteria by phase for patient-reported outcome measure-development across SLE-PRO measures

	LupusQoL	LupusQoL-US	LupusPRO v1.7	LupusPRO v1.8
*Adequacy of item generation process*				
Derivation of items is appropriate	The study population’s representativeness of the target population is questionable with no evidence to support semi-structured nature of interviews	Limitations exist; did not conduct concept elicitation for target population as content validity was assumed	The study population’s representativeness of the target population is questionable with no evidence to support semi-structured nature of interviews	Limitations exist; did not conduct concept elicitation for target population as content validity was assumed
Derivation of items is comprehensive	No evidence that concepts affected majority of patients representing the target population or whether a wide range of patients representing target population were used. No evidence of achievement of saturation, except statement in text (e.g., no saturation grid)	Limitations exist; did not conduct concept elicitation for target population as content validity was assumed	No evidence that concepts affected majority of patients representing the target population or whether a wide range of patients representing target population were used. No evidence of achievement of saturation, except statement in text (e.g., no saturation grid)	Limitations exist; did not conduct concept elicitation for target population as content validity was assumed
*Adequacy of cognitive interviewing process*				
Cognitive interviewing with patient input	Limitations exist concerning the representativeness of the study population to the target population and insufficient documentation of details	Limitations exist; did not conduct cognitive debriefing for target population as content validity was assumed	Limitations exist; target population and documentation of details	Limitations exist; did not conduct cognitive debriefing for target population as content validity was assumed
Cognitive interviewing until saturation is reached	No evidence of achievement of saturation, except statement in text (e.g., no saturation grid)	Limitations exist; did not conduct cognitive debriefing for target population as content validity was assumed	Limitations exist; target population and insufficient documentation of details	Limitations exist; did not conduct debriefing for target population as content validity was assumed
*Testing of other psychometric properties*				
Testing of items/concepts	Limitations exist for the “target population” component of development Transparency lacking	Limitations exist for the “target population” component of development. Transparency lacking	Limitations exist for the “target population” component of development Transparency lacking	Limitations exist for the “target population” component of development. Transparency lacking

**Table 4:** Summary of data abstraction and assessment notes for measurement properties and documentation using evaluation criteria by phase for patient-reported outcome measure-development across SLE-PRO measures

	LupusQoL	LupusQoL-US	LupusPRO v1.7	LupusPRO v1.8
*Content validity*				
Item-generation	Limited details, lacking transparency; Evidence not sufficient to support measure adequacy	Limited details, lacking transparency; Evidence not sufficient to support measure adequacy	Limited details, lacking transparency; Evidence not sufficient to support measure adequacy	Limited details, lacking transparency; Evidence not sufficient to support measure adequacy
Cognitive interviewing	Limited details, lacking transparency	Limited details, lacking transparency	Limited details, lacking transparency	Limited details, lacking transparency
*Other psychometric property testing*				
Reliability	Available	Available	Available	Available
Construct validity	Available	Available	Available	Available
Ability to detect change	Available	Not available	Available	Not available

### ***LupusQoL*** [15, 31]

The evidence is insufficient for the target population, concepts measured, measurement properties, and documentation. Of the “No” responses determined from data abstraction, three of the four “No” responses were questions with an asterisk for the target population component. Similarly, two of four questions with an asterisk were deemed “No” for concepts measured. Overall, evidence is not sufficient to support measure adequacy in terms of content validity phase for measurement properties. The documentation of the instrument development process is insufficient with details lacking to reproduce the study, including item generation and cognitive debriefing phases of development.

### ***LupusQoL-US*** [16]

The evidence is insufficient for the target population, concepts measured, measurement properties, and documentation. Likewise, three of the four “No” responses were key questions for the target population evaluation.

Furthermore, the evidence for concepts measured is insufficient because item-generation was not conducted. The evidence is not sufficient to support measure adequacy in terms of content validity for the measure properties component of the evaluation. The review of reliability and construct validity were deemed available for the instrument as testing details and results were reported. Ability to detect change was not reported. The documentation of the instrument development process is insufficient due to lack of transparency across phases and limited documentation.

### ***LupusPROv1.7*** [17]

The evidence is insufficient for the target population, concepts measured, measurement properties, and documentation. Three of the four “No” responses were key questions for the target population evaluation.

The evidence is insufficient for concepts measured in terms of content validity. Similarly, the evidence is not sufficient to support measure adequacy in terms of content validity for measurement properties. The evaluation of reliability, construct validity, and ability to detect change were deemed available for the instrument as testing details and results were reported. The documentation of the instrument development process is insufficient due to limited details on processes and supporting evidence.

### ***LupusPROv1.8*** [18]

The evidence is insufficient for the target population, concepts measured, measurement properties, and documentation. Three of the four “No” responses were key questions for the target population evaluation.

The evidence is insufficient for concepts measured because item-generation and cognitive testing was not conducted. Additionally, the evidence is not sufficient to support measure adequacy in terms of content validity for measurement properties. On another note, the evaluation of reliability and construct validity were deemed available for the instrument as testing details and results were reported. Ability to detect change was not reported. The documentation of the instrument development process is insufficient.

## Discussion

To our knowledge, this study represents the first review of the most widely used SLE-PRO measures to assess how well they align with the recommendations of FDA 2009 PRO guidance. Our results contradict our hypothesis that PRO measures developed after the FDA 2009 PRO guidance release would be adherent (or more adherent) to the FDA recommendations than those developed prior to 2009. In fact, our review found mixed results regarding alignment with FDA-guidance recommendations regarding target population, concepts measured, testing of other psychometric properties, and documentation for all the measures examined. Some or much of this misalignment may be due to lack of availability of the detailed documentation on development needed to assess if the FDA guidance was followed.

The LupusQoL and LupusPRO SLE-PRO have been used for many years and have led to many advancements in capturing what is most important to patients with SLE. For the original SLE instruments, the evaluation of concepts measured involved patient-engagement interviews with concepts elicited until saturation. Moreover, cognitive testing allowed for patients to provide input on the draft versions of the measures. Documentation of the development and validation process was enhanced with figures depicting that process, as well as identified domain structures. Despite these strengths, important limitations were identified in our assessment. Often, due to not finding any information or lack of sufficient detail in the documentation identified.

To date, awareness of PRO guidance recommendations is unknown in research settings outside of the pharmaceutical industry (e.g., clinical trials vs clinical care). We postulate that some PRO measures may not align with FDA guidance because there is lack of knowledge about the guidance in some sectors with possible reliance on checklists [32, 33] and a lack of understanding on how to execute and evaluate the processes described in the guidance. This might explain why some developers cite the FDA 2009 guidance, but do not align with recommendations. As an example, the Engelberg Center for Health Care Reform at the Brookings Institution published a report discussing opportunities and challenges in the development and use of PROs [34]. The report summarized experiences gathered from an expert workshop across five sessions discussing challenges with FDA PRO guidance.

While the LupusQoL and LupusPRO measures were not developed in the context of clinical-trial use for product approval and labeling claims, acknowledgement of FDA guidance was noted by the developers [17, 31]. Yet, not, all processes and/or level of documentation are aligned with FDA guidance recommendations. For all measures (original and revised), the target population is unclear as study population characteristics varied, were not consistently reported, or were not considered across the item-generation, cognitive-testing, and other psychometric-testing phases. The information available on development is limited and lacking detail on the qualitative processes. For example, the original measure-development work engaged patients in the development process, but documentation on content validity was not detailed enough to understand if/how it aligned with the guidance. It is unclear if a wide range of patients representing the target population were interviewed and whether the concepts were experienced by the majority of sample population. Additionally, there was not documentation that indicated items were developed using the exact words as described by patients in the interviews, nor documentation from the testing of item wording. Similarly, documentation confirming item response options, the recall period of the measure, etc., were not available. These findings are similar to other reports regarding PRO labelling claims rejected by FDA for lack of content validity as well as a systematic review evaluating qualitative methods used to generate instruments [10, 11, 35]. Similarly, developers may have learned of FDA guidance after development. For example, McElhone et al., developed the LupusQol prior to release of the FDA guidance and published an analysis on ability to detect change in 2016, citing the FDA guidance in the evaluation [31].

Another issue may be terminology used in identified reporting that may not have been clear. For example, content validity typically encompasses both item generation and cognitive interviewing. However, the original measures appear to have had content validity assessed through cognitive interviewing only. Similarly, face and content validity terms were used interchangeably. Face validity is evaluated after an instrument has been developed whereas content validity is embedded in the development process [36]. Documentation also was lacking to determine if saturation of concepts was reached or deemed comprehensive, as well as whether the potential for bias in interviewing for concept elicitation or cognitive debriefing was mitigated. For example, interviewing should be conducted using open-ended questions in contrast to directed questions that can be answered simply with yes/no response.

Documentation of instrument origination may enhance understanding of the rationale behind decisions made during the developmental process. Documentation provides transparency and evidence in support of preliminary instrument development, content validity, measure development, interpretation, as well as any changes made to the measure. Otherwise, decisions may not be clear to potential users seeking permission to use PRO instruments. An example is highlighted by Mathias et al., who argued in their 2018 study that the recall period of existing instruments did not capture accurate reporting of fluctuations in SLE symptoms and impacts of the disease [29]. As a result, a 24-hour (h) recall period would be more appropriate for all symptoms except hair loss in contrast to the conventional 4 weeks. The suggested 24-h recall period was confirmed by patients as they reported daily fluctuations [29]. Documentation allows reviewers to understand methodology and evaluate if data generation processes were suitable and complete for the target population (e.g., the identification and inclusion of concepts that matter most to patients). The documentation process applies for disease-specific and disease-agnostic measures, including legacy measures. Others can contribute to the literature by expanding upon and carrying the documented instrument forward while minimizing redundancy. Not only is documentation important in the development process, but it is also important when making modifications to existing instruments. Existing instruments may be modified when administered in RCTs, however, the modifications are not transparent nor tested [37]. To assist with the incorporation and qualification of PRO measures in RCTs, Coles et al. [38] proposed the development of a publicly available validity repository of "validity arguments", as a mechanism to collect evidence to support the validity of PRO measures respective to the context of use.

The FDA PFDD guidance series is underway to provide more detail on development of COAs for use in regulatory approval of medical products. With the pending draft and final releases of the FDA PFDD guidance series, the 2009 guidance remains in effect. Not only will appropriate use of these documents improve transparency of the development process, consistency in selection of the study population across development and/ or testing phases, and engaging patients appropriately when adaptation existing PRO measures, but also for newly developed measures. Effective use of more detailed PRO guidance may improve standardization of the process and documentation, thereby raising uptake in the use of PRO measures due to comparability and enhanced understanding in interpretation of results. Adherence to FDA guidance will increase the chances of FDA accepting COA tools as fit-for-purpose (e.g., FDA Drug Development Tools COA Qualification Program: https://www.fda.gov/drugs/clinical-outcome-assessment-coa-qualification-program/clinical-outcome-assessments-coa-qualification-program-resources). This is imperative as PROs can provide a comprehensive view of the patient experience in patient-focused drug development and related research. As previously mentioned, LupusPRO has not been used in RCTs, while the LupusQoL was used in three randomized, controlled trials with scores being used as exploratory endpoints [19–21]. To note, the review by Izadi et al., from 2018 highlighted that LupusQoL had been used in one RCT, however, data was not provided [19]. This may be the reason the RCT was excluded in newer reviews and therefore, was not included as part of the RCTs mentioned above [20, 21]. If PRO data is not deemed fit as a primary endpoint due to nature of the study, having PRO data act as secondary endpoints can support primary endpoint interpretation. In the 2018 review by Mercieca-Bebber et al., there are several examples of how PRO data used as primary or secondary endpoints contributed to approval of treatments [5].

It is recognized that the reviewed documents may be providing limited insight into the development and validation processes. Developers may have followed FDA guidance for PRO development and validation processes but did not document or describe detail adequately to demonstrate evidence of alignment. Under these circumstances, our review is limited, as we are only able to evaluate documents that are publicly available and accessible. Furthermore, developers’ perceptions and interpretation of FDA guidance may differ compared to that of others. Based on our review, developers should ensure: patient involvement in the process; that the study population characteristics are similar across all phases of measure development; and clear and publicly available documentation of all methods. The FDA advocates for documentation of development process to be made publicly available and accessible, including—but not limited to—cognitive interview summaries or transcripts, source of items, and an item-tracking matrix. “Without adequate documentation of patient input, a PRO instrument’s content validity is likely to be questioned” [6]. Publication limitations means authors need to consider using an appendix or supplementary materials section to make those details available. Alternatively, authors can make the information available in an accessible users-manual [6].

## Conclusions

Despite developers’ original efforts to establish content validity and other measurement properties, limitations identified here make it unclear for which target population(s) the current Lupus measures are fit-for-purpose. For the development of fit-for-purpose COAs, our results indicate a need for improvement in awareness and understanding of FDA guidance, this includes the role of patients in development and importance of detailed documentation to support the measure’s content validity. With the pending draft and final releases of the new FDA PFDD guidance series, further work will be needed to enhance awareness and appropriate use of these documents.

## Supplementary Information

**Additional file 1.** **Appendix 1.** Instructions on Using the Evaluation Criteria. **Appendix 2.** Brief Summaries of Instrument Details. **Appendix 3.** Instrument Evaluation Criteria Summaries.

**Additional file 2.** **Table S1.** Food and Drug Administration's (FDA) 2009 Patient-Reported Outcome (PRO) Guidance Terms and Definitions Used to Develop Evaluation Criteria.

**Additional file 3.** **Table S2.** Overview of Evaluation Criteria for Key Components by Phase for Patient-Reported Outcome Measure-Development.

## Data Availability

The data used and/or analyzed are available from the original publications as well as secondary publications online.
