# Supplementary material for: Capturing the patient experience in systemic lupus erythematosus: Are widely used measures fit-for-purpose and adherent to FDA PRO guidance recommendations?
Source: J Patient Rep Outcomes. 2022 Jan 21;6:7. doi: 10.1186/s41687-022-00411-8 (PMC8777546; doi:10.1186/s41687-022-00411-8)
Supplement: Supplementary file 1 — Additional file 1. Appendix 1. Instructions on Using the Evaluation Criteria. Appendix 2. Brief Summaries of Instrument Details. Appendix 3. Instrument Evaluation Criteria Summaries. [file 41687_2022_411_MOESM1_ESM.docx]

**Supplementary Material**

Supplement to: *Capturing the patient experience in systemic lupus erythematosus: Are widely used measures fit-for-purpose and adherent to FDA PRO guidance recommendations?*

**Contents:**

Appendix 1. Instructions on Using the Evaluation Criteria…………………………………………………… Page 2

Appendix 2. Brief Summaries of Instrument Details…………………………………………………………Pages 3-4

Appendix 3. Instrument Evaluation Criteria Summaries……………………………………………………. Pages 5-9

**Appendix 1. Instructions on Using the Evaluation Criteria for Key Components of Patient-Reported Outcome Measure Development and Testing**

*A user may not need to answer all questions if a previous response is indicated as “No”:*

- If the response to a question with an asterisk * is “No”, it is not necessary to continue with the evaluation.
- If the response to a question with an asterisk * is “No” for *Target Population*, *Concepts Measured, or Measurement Properties*, content validity is questionable. As a result, additional testing (e.g., other psychometric property testing) is irrelevant and evaluation of other key concepts, i.e., *Measurement Properties* and *Documentation* is not necessary.

*Determination of sufficient/insufficient evidence for key components:*

- Target Population: If any of the *Target Population* questions with an asterisk * are indicated as “No”, the evidence is insufficient, questions 1-6.
- Concepts Measured: If any of the *Concept Measured* questions with an asterisk * are indicated as “No”, the evidence is insufficient.
  - Content validity encompasses questions 7-10.
    - Item generation: Q7,8
    - Cognitive interviewing: Q9,10
  - Other psychometric property testing encompasses questions 11. Note, this question is required for evidence.
- Measurement Properties: If any of the *Measurement Properties* questions with an asterisk * are indicated as “No”, the evidence is insufficient for item generation and cognitive interviewing. Questions 15-17 for other psychometric property testing phase are deemed as “Available” or “Not Available” based upon “Yes” or “No” responses, respectively.
  - Content validity encompasses questions 12-17.
    - Item generation: Q12,14
    - Cognitive interviewing: Q13,14
  - Other psychometric property testing encompasses questions 14, 15-17.
- Documentation: If any of the *Documentation* questions are indicated as “No”, the evidence is insufficient for the corresponding phase of development, questions 18-25.
  - Content validity encompasses questions 18-24.
    - Item generation: Q19, 21, 22, 23
    - Cognitive interviewing: Q20, 21, 24
- Other psychometric property testing encompasses questions 21, 25, 26.

**Appendix 2. Brief Summaries of Instrument Details**

LupusQoL

The LupusQoL contains 34-items across eight domains (physical health, emotional health, body image, pain, planning, fatigue, intimate relationships, and burden to others) and was developed with the psychometric properties tested in British adults with SLE. ^15^ Scoring is based on a 5-point Likert scale (0 = all the time to 4 = never). Domain scores are totaled and divided by the number of items within the domain and multiplied by 100. Mean domain scores are transformed to scores ranging from 0 to 100 with higher scores indicating better health. The recall period is four weeks. Time for completion takes less than 10 minutes. Overall Cronbach’s alpha was not available. Cronbach’s alpha coefficients for the domains ranged from 0.88 to 0.96. Discriminant validity was demonstrated for varying levels of disease activity and damage. Concurrent validity was demonstrated on four comparable domains of the SF-36 ranging from r=0.71-.79.

LupusQoL-US

LupusQoL-US, adapted from LupusQoL for the US ethically heterogeneous population with SLE, contains 34-items with five domains (physical health/pain/planning, intimate relationships, burden to others, emotional health/body image, fatigue).^16^ No deviations in scoring were reported as the measure contains the same number of items as compared to the original LupusQoL. Language modifications were performed using feedback from patient interviews. The recall period is four weeks. Cronbach’s alpha coefficients for the original eight domains ranged from 0.85 to 0.94. Discriminant validity was demonstrated for varying levels of disease activity, except for the pain, intimate relationships, body image, and fatigue domains as well as varying levels of disease damage, except for the intimate relationships and fatigue domains. Concurrent validity was demonstrated on comparable domains of the SF-36 ranging from r=0.54-0.73.

LupusPROv1.7

LupusPROv1.7 contains 44-items across nine HRQoL domains and four non-HRQoL domains (n=13 total domains if pain-vitality is combined) and was developed and psychometrically tested for use in the ethnically heterogeneous population with SLE in the US.^17^ The HRQoL-related domains include lupus symptoms, physical health, pain-vitality, emotional health, body image, cognition, procreation, and lupus medications, and general health. The non-HRQoL domains include available social support, coping, desires-goals, and satisfaction with medical care. Scoring is based on a 5-point Likert scale (0 = none of the time/not applicable to 4 = all the time). Reverse scoring for some items is required. Domain scores range from 0 to 100 with higher scores indicating better health. The recall period is 4 weeks. Time for completion takes less than 10 minutes HRQoL and non-HRQoL scores may be calculated by averaging the respective domains. The Flesch reading ease (acceptable range 60-70) and Flesh-Kincaid grade level (seventh grade reading level) was evaluated and within range with 68.7 and 6.7, respectively. Cronbach’s alpha coefficients for the domains ranged from 0.68-0.94. Concurrent validity was demonstrated on comparable domains of the SF-36 ranging from r=-0.26-0.75. Criterion validity was demonstrated with domains and scores correlated with disease activity and damage measures.

LupusPROv1.8

LupusPROv1.8, a revised version of LupusPROv1.7, was modified to separate the pain-vitality domain into sleep, pain, and vitality and psychometrically tested for use in the ethnically heterogeneous population with SLE in the US.^18^ The LupusPROv1.8 publication states that the v1.7 version contained 43-items (instead of 44-items as reported in v1.7 publication), and therefore, v1.8 contains 49 items with six items added. As it is not explicitly stated in the original publication, it is assumed the general health domain (i.e., question asking participants to rate their own health) was removed or is no longer considered a part of the questionnaire. As a result, v1.8 contains a total of n=14 domains. The HRQoL domains include lupus symptoms, physical health, sleep, pain, vitality, emotional health, body image, cognition, procreation, and lupus medications. The non-HRQoL domains include social support, coping, desires-goals, and satisfaction with medical care. No deviations in the recall period, time for completion, or scoring from the original measure were reported. Cronbach’s alpha coefficients for the domains ranged from 0.68-0.97, overall HRQoL 0.96, and overall non-HRQoL 0.81. Concurrent validity was demonstrated on comparable domains of the SF-36 ranging from r=-0.27-0.94.

**Appendix 3. Instrument Evaluation Criteria Summaries**

LupusQoL^15,25^

Target Population: The intended target-SLE population was British adults with SLE. The characteristics of the target population varies across phases of development, and characteristics are not reported for all phases. Without documentation of study population characteristics, evidence is not sufficient.

Concepts Measured: The items contained in LupusQoL were derived from both the use of semi-structured face-to-face interviews with patients as well as experts, but no documentation was found to support the semi-structured nature of the interviews. The authors stated that interviews were conducted with concepts elicited without any details on process for concept elicitation to ensure it was comprehensive. A draft instrument was reviewed and discussed among a panel of experts. Cognitive interviewing of the draft instrument was assessed via patient feedback, with limited documentation of details., e.g., revisions to the draft instrument. The evidence is insufficient as details in methodology and results were lacking to determine if concepts were comprehensive and relevant.

Measurement Properties: Overall, evidence is not sufficient to support measure adequacy in terms of content validity. Males did not participate in item generation or cognitive interviewing, however, males commented on later stages of instrument development. Similarly, Blacks were not represented in item generation. Population characteristics were not consistently reported; ethnicity, education, and SLE duration were not reported for other psychometric testing. Disease activity and disease damage were not considered across item generation, cognitive interviewing, or other psychometric testing phases. The authors stated that interviews were conducted with concepts elicited until saturation without details on the process for concept saturation. Cognitive interviewing of the draft instrument was assessed via patient feedback, with limited documentation of details., e.g., saturation during the cognitive interviewing process. In contrast, the evaluation of reliability, construct validity, and ability to detect change were deemed available for the instrument as testing details and results were reported. Despite not been assessed during the initial 2007 development, the developers evaluated the measure’s ability to detect change with their 2016 publication referencing the FDA guidance, however, no comments on modifications or future improvements were made.^25^

Documentation: The documentation of the instrument development process is insufficient. A diagram within the publication depicts the stages of development and validation as well as the number of items removed throughout the stages, thus making an attempt at transparency throughout the development process. The recall period was selected to resemble the British Isles Lupus Assessment Group (BILAG) index, however, patients’ feedback was not provided.^15^ Details are lacking with regards to the characteristics of patients who tested the draft instrument and provided comments about the design, content, structure, and response scale.^15^ In addition, transparency was lacking in revisions, e.g., evidence of patient feedback to ensure understandability and completeness of items.

LupusQoL-US^16^

Target Population: The evidence is insufficient across phases of development. The intended target-SLE populations were US adults with SLE and it is important to note, that the instrument was not developed for a designated clinical trial. The study population characteristics were not available for all phases of development, resulting in deeming the evidence insufficient for use in the intended population.

Concepts Measured: The evidence is insufficient because item-generation was not conducted. LupusQoL-US “assumed content validity was present, as the items were generated based on patient feedback” and “items and their integrity were maintained in the modification process of the UK version”.^16^ Cognitive interviews obtained patient input on wording of items. Wording of items were revised using a team of reviewers and developers of the original instrument with additional cognitive testing and revisions performed until no further suggestions. FA of LupusQoL-US identified five HRQoL domains using EFA, as the CFA of eight domains resulted in a poor fit.

Measurement Properties: Overall, the evidence is not sufficient to support measure adequacy in terms of content validity. The lack of patient input for the comprehensiveness of items in adapting the instrument from UK to US SLE patients deemed content validity inadequate. The authors acknowledge this limitation in the discussion section of the original LupusQoL-US publication noting that important patient concerns may be missing; the US is ethnically heterogeneous compared to the UK. The evaluation of reliability and construct validity were deemed available for the instrument as testing details and results were reported. Ability to detect change was not reported.

Documentation: The documentation of the instrument development process is insufficient. A diagram within the publication depicts the modification process of the original instrument as well as the domain structures of the UK and US versions. In addition, examples of feedback from the cognitive testing in terms of wording changes were provided (e.g., “slower pace” replaced with ‘do things more slowly”). To enhance transparency, FDA guidance recommends an item tracking matrix or similar documents to be utilized for instrument revisions or patient feedback to ensure understandability and completeness of items. Moreover, having study population characteristics of the patients who participated in the cognitive testing would be helpful to better understand the population used for content validity. Documentation of other psychometric property testing varied in terms of transparency and clarity. It is unclear if the LupusQoL-US structure is based on the eight or five domains as other psychometric testing utilized the eight domains (i.e., testing of reliability and construct validity).^16^ Moreover, transparency in the proposed methods and analysis may be improved with more detail prior to reaching the results section to build a complete picture of methods and analysis. Sufficient details were provided in other areas such as correlation interpretation, proposed sample size, and FA. FDA PRO guidance was not cited, however, the developer who created LupusPRO cited FDA guidance in the LupusPRO publication.

LupusPROv1.7^17^

Target Population: The evidence is insufficient across phases of development. The intended target-SLE populations were ethnically heterogeneous US adults with SLE. As such, the instrument was not developed for a designated clinical trial. The developers state LupusPRO may be used as a screening tool in the clinic setting, but study population characteristics during development are lacking and thereby not providing sufficient evidence for use in the intended population.

Concepts Measured: The evidence is insufficient. The items contained in LupusPROv1.7 were derived using a semi-structured approach, but evidence is needed to support the semi-structured nature of the interviews. Interviews and item-pool creation were directed by literature, existing tools, and input from clinicians. The draft instrument was tested among patients regarding the “importance” and “bothersome” via a clinometric feedback method.^17^ Additional pretesting and other psychometric testing were performed followed by revisions and cognitive interviewing to have the finalized version psychometrically tested in patients from multiple centers.

Measurement Properties: Overall, the evidence is not sufficient to support measure adequacy in terms of content validity. The developers discuss the small sample size for item generation as a limitation but believe saturation of concepts was achieved. The patient engagement interviews were conducted with concepts elicited until saturation without any details on process for concept saturation. Aside from documentation which will be discussed below, LupusPROv1.7 had ethnicity characteristics differ across phases of development without reporting population characteristics for cognitive testing of the instrument. The evaluation of reliability, construct validity, and ability to detect change were deemed available for the instrument as testing details and results were reported.

Documentation: The documentation of the instrument development process is insufficient. Transparency may be improved in the development and validation processes by using consistent and appropriate use of terminology used in FDA guidance. For example, the content validity process was unclear as terminology among researchers may differ (e.g., “cognitive interviewing” when referring to item generation and “content validity” when referring to cognitive testing). The methodological process was challenging to understand regarding the number of domains identified as the abstract, conceptual model/methods, and results section suggest different domain structures; the abstract identifies 11 domains. It may be assumed that the initial instrument structure was represented by the conceptual model of domains as a hypothesized framework, however, confusion stems from the results indicating the PCA resulted in six domains; whereas reliability and validity table results indicate n=13 domains. On another note, further clarity regarding the sources of patient recruitment for the initial phases of development prior to the psychometric analyses may be helpful in better understanding the patient population. Cognitive interviewing was assessed via patient feedback with limited item-tracking documentation. The v1.8 publication states that the v1.7 utilized the FDA PRO guidelines for development. The development process may be better understood with additional details (e.g., saturation grid) similar to what is presented in the publication, e.g., the diagram depicting the development process as well as the conceptual framework for the hypothesized domain structures.

LupusPROv1.8^18^

Target Population: The evidence is insufficient across phases of development. The intended target-SLE populations were ethnically heterogeneous US adults with SLE, and the instrument was not adapted for a designated clinical trial. The study population characteristics were not available across all phases of development and could not provide evidence for use in the intended population.

Concepts Measured: The evidence is insufficient because item-generation and cognitive testing was not conducted. LupusPRO v1.8 did not re-assess content validity as “LupusPRO was derived from SLE patients, which established its face validity”.^18^ The updated LupusPRO v1.8 instrument added six items and separated the original pain-vitality domain into three distinct domains; pain, vitality, sleep for patient care and clinical trials.

Measurement Properties: The measure assumed content validity. The evidence, however, is not sufficient to support measure adequacy in terms of content validity (Table 5). Six items were added to the measure; however, cognitive interviewing of the items was not conducted and thus, leaving readers to question if items were derived from v1.7 and previously tested. LupusPROv1.8 differed on ethnicity and education characteristics when compared to the original version. On another note, the evaluation of reliability and construct validity were deemed available for the instrument as testing details and results were reported. Ability to detect change was not reported.

Documentation: The documentation of the instrument development process is insufficient. A diagram within the publication depicts the identified domain structures. However, confusion regarding the number of items was previously discussed above. The population sample size is not clear as the results section from the publication reported n=131 whereas the table reports n=137. Moreover, limited details were provided regarding the six items added to v1.8 (e.g., if items were originally removed from the v1.7 testing).
