# Supplementary material for: Capturing the patient experience in systemic lupus erythematosus: Are widely used measures fit-for-purpose and adherent to FDA PRO guidance recommendations?
Source: J Patient Rep Outcomes. 2022 Jan 21;6:7. doi: 10.1186/s41687-022-00411-8 (PMC8777546; doi:10.1186/s41687-022-00411-8)
Supplement: Supplementary file 2 — Additional file 2. Table S1. Food and Drug Administration's (FDA) 2009 Patient-Reported Outcome (PRO) Guidance Terms and Definitions Used to Develop Evaluation Criteria. [file 41687_2022_411_MOESM2_ESM.docx]

| **Supplementary Table 1.** Food and Drug Administration’s (FDA) 2009 Patient-Reported Outcome (PRO) Guidance Terms and Definitions Used to Develop Evaluation Criteria^6^ | |
| --- | --- |
| **Terms** | **FDA 2009 PRO Guidance Definition** |
| **Target Population** | The intended patient population studied in the PRO measure-development process. |
| **Concepts Measured** | The specific measurement goal (i.e., the thing that is to be measured by a PRO instrument). PRO concepts represent aspects of how patients function or feel related to a health condition or its treatment. |
| **Measurement Properties** | All the attributes relevant to the application of a PRO instrument including content validity, construct validity, reliability, and ability to detect change. These attributes are specific to the measurement application and cannot be assumed to be relevant to all measurement situations, purposes, populations, or settings in which the instrument is used. |
| **Documentation** | Instruments and related development history should be available and accessible publicly. If development history is not available, provide documentation of content validity with an application including open-ended patient input from the appropriate population.  -Development process should reveal the means by which the items and domains were identified including literature review, expert input, and qualitative research.  -The exact words used to represent the concepts measured by domain or total scores should be derived using patient input.  -Complete list of items generated and the reasons for deleting or modifying items.  -Development and testing will be reviewed to determine that the items cover all aspects of the concept important to patients, and saturation has been reached. |
| **Content validity** | Evidence from qualitative research demonstrating that the instrument measures the concept of interest including evidence that the items and domains of an instrument are appropriate and comprehensive relative to its intended measurement concept, population, and use. Testing other measurement properties will not replace or rectify problems with content validity. Documentation to support content validity should include literature review and expert input; all item generation techniques used, including any theoretical approach; the populations studied; source of items; selection, editing, and reduction of items; cognitive interview summaries or transcripts; pilot testing; importance ratings; and quantitative techniques for item evaluation. |
| **Item Generation/**  **Cognitive Interviewing** | Includes input from the target patient population to establish the items that reflect the concept of interest and contribute to its evaluation. Item generation generally incorporates the input of a wide range of patients with the condition of interest to represent variations in severity and in population characteristics such as age, sex, ethnicity, and language groups.  Cognitive interviewing may aid with assessing patient understanding of the items being asked in the instrument. This includes item wording, completeness of items, clarity, and readability. Content should be reviewed for literacy level, questions patients may be unwilling to answer, formatting, instructions, time required, and font size. |
| **Reliability** | The ability of a PRO instrument to yield consistent, reproducible estimates of true effect. |
| **Construct validity** | Evidence that the relationships among items, domains, and concepts conform to a priori hypotheses concerning the logical relationships that should exist with other measures or characteristics of patients and patient groups. Includes convergent, discriminant, known-groups validity. |
| **Ability to detect change** | Evidence that a PRO instrument can identify differences in scores over time in individuals or groups who have changed with respect to the measurement concept. |
