# Supplementary material for: Capturing the patient experience in systemic lupus erythematosus: Are widely used measures fit-for-purpose and adherent to FDA PRO guidance recommendations?
Source: J Patient Rep Outcomes. 2022 Jan 21;6:7. doi: 10.1186/s41687-022-00411-8 (PMC8777546; doi:10.1186/s41687-022-00411-8)
Supplement: Supplementary file 3 — Additional file 3. Table S2. Overview of Evaluation Criteria for Key Components by Phase for Patient-Reported Outcome Measure-Development. [file 41687_2022_411_MOESM3_ESM.docx]

| **Supplementary Table 2** Overview of Evaluation Criteria for Key Components by Phase for Patient-Reported Outcome Measure-Development | | | |
| --- | --- | --- | --- |
|  | **Phase of Development** | | |
|  | **Content Validity** | **Other Psychometric Property Testing** | **Modifications** |
|  | Phase was represented by:  Item generation  Cognitive Interviewing | Phase was represented by:  Reliability  Construct Validity  Ability to Detect Change | Phase was represented by:  Re-evaluation of content validity and/or other measurement properties to provide evidence of instrument adequacy |
| **Key Components** |  |  |  |
| **Target**  **Population** | Evidence can be identified from documentation for the patient population of interest. This refers to the patients that this instrument will be designed for/who will complete it. The patient population is described in terms of condition characteristics (e.g., condition, severity, etc.) and demographic characteristics (e.g., age, gender, race, education level). The target population was transparent and clearly specified. | Evidence can be identified from documentation of testing the measure in the patient population that was used to develop the measure (e.g., age, gender, race, education level, condition severity, etc.). | Supporting rationale is provided for adapting to a different population/culture than what was originally intended. |
| **Concepts Measured** | Initial evidence from literature review and documentation of expert input. Additional evidence can be identified from documentation that the qualitative interviews obtained patient input regarding the generation of new items. Item generation was appropriate and comprehensive (e.g., interview transcripts demonstrating open-end questions and items derived from the transcripts across a range of patients from the target population).  Evidence can be identified from documentation that the qualitative interviews provided the patients’ interpretation of items and terms and ensured understanding and completeness of the concepts contained in the items (interview transcripts to evaluate patient understanding) were clearly described. | Evidence can be identified from supporting documentation to confirm concepts with scoring rule and reduction of items through appropriate testing. | Evidence can be identified from documentation of modifications, i.e., response options, wording, creation of new items. |

| **Table 2.** (continued) | | | |
| --- | --- | --- | --- |
| **Measurement Properties** | Evidence is provided demonstrating the study population is representative of the target population and the population openly provides input until saturation is reached. Input provides the basis for confirmation or need for revisions of the draft instrument development  in terms of items, mode, frequency, duration, and intensity/burden of admin, instructions, etc. | Evidence is provided demonstrating reliability: stability of scores over time when no change is expected; construct validity (convergent, discriminant, known-groups): relationship among items, domains, and concepts conform to hypotheses; ability to detect change: group and within-person change over time. | Supporting documentation demonstrates stability of scores over time when no change is expected, relationship among items, domains, and concepts conform to hypotheses, as well as group and within-person change over time. |
| **Documentation** | Evidence is provided supporting the development of draft instrument including information about the target population and study population, concepts in terms of item generation, cognitive interviewing, measurement properties of content validity, e.g., transcripts, saturation grid, item-tracking matrix, etc. Furthermore, the selection of recall period, response options, and format were clearly described. | Evidence is available reporting of measure development, scoring and psychometric testing, and reporting of results including information on respondent participating in pilot testing/completing survey (i.e., study population). | Supporting documentation is available for measure adaptation/modifications are based on documentation requirements for content validity and other measurement property assessments. |
